# On a New Theory of Vision

**Published:** 1863-01

**Authors:** J. Alexander Davies


### Art. VII.—ON A NEW THEORY OF VISION, RESULTING FROM A NEW THEORY OF THE HUMAN MIND.

By J. Alexander Davies.

The doctrine I am about to explain I have, according to the distinction of Dr. Brown, which I take to be a philosophical one, called a *theory.* Concerning the difference between an hypothesis and theory, he has said (*Phil. Hum. Mind,* Lect. 8), "We commonly give the name of hypothesis to cases in which we suppose the intervention of some substance, of the existence of which, as present in the phenomenon, we have no direct proof, or of some additional quality of a substance before unobserved; and the name of theory to cases, which do not suppose the existence of any substance that is not actually observed, or of any quality that has not been actually observed, but merely the continuance, in certain new circumstances, of tendencies observed in other circumstances"—a passage I have thought it would be of utility to adduce, the distinction in question being very commonly overlooked. The *theory,* then, which I would set forth is founded upon, and manifestly follows from, a new theory concerning the nature of the human mind, (both the intellectual and moral nature), which may thus be stated. Without denying the possibility either of the present or future existence of matter, of which the perception would be possible without that of length and breadth—or, as it is generally said, not *having* length and breadth, the absence of which conditions constitutes the idea of spiritual substance, that is to say, the knowledge of what would form this idea—it may be affirmed that we cannot conceive such a possibility. On this account, however, it would not be philosophical either to make the denial in question, or, perhaps, doubt the possibility; but, according to the law of Newton, not to look for more causes than are necessary to explain the known effects, it may properly be enquired whether the absence of the above-mentioned conditions *is* necessary to account for the phenomena of the human mind. I believe that it is not.

To every previous material hypothesis or theory of the human mind, it may be objected that no reply has been given to the possible enquiry—*What* part of the material substance supposed to be the mind reflects, remembers, imagines, and feels?—an objection which the ingenious Priestley did not answer, and therefore, undoubtedly, did not anticipate. It is, however, a real one; whereas, the notion that matter cannot think, is only a prejudice. It must be allowed that, possibly, this notion is true, nevertheless, it is believed only from prejudice—those believing it not being able to give any philosophical reason for their doing so. The supposition that the mind is the *result* (combination or aggregate) of the *forces* of the *various* parts composing it, is not liable to this objection, and one which has parallels in physical science. According to it the *several parts* of the mind are *not* the mind itself, this being the result of the combination or union of their respective forces; so that it cannot be enquired, *what* part of the substance composing it thinks, or otherwise sustains, whether actively or passively, a function of mind, or itself. Similarly, the magnetism produced by electricity is the result of action which is *not* magnetic, and electricity accrues from motion, the friction of certain bodies, which motion is *not* electric; and so also the light consequent upon friction and galvanism, proceeds from motions which are *not* luminous. In a word, physically, we find the development (whether by the term be understood the transformation or creation) of force, on which account the possibility of the truth of this psychological theory—so called because the existence of no new laws is supposed—cannot be denied; for it is of like character with the cases referred to, and by analogy is rendered probable. It cannot, indeed, be denied, because of this similarity—the proposition being that mind results from various forces, none of which are *in any degree* mental.

Having thus briefly stated what I consider to be a new theory of the mind, I shall now show how the theory of vision which I am wishful to propose—a theory, indeed, which may be applied to every sense—follows from it. All the attempts which have been made to explain the cause of erect vision have been founded upon the supposition that the mind perceives objects *positionally*—a supposition, however, which not only has not been proved to be true, but is inconsistent both with the doctrine I have stated, and that of the mind being an unextended substance; inasmuch as an object manifestly cannot be impressed positively upon a mind whose *component* parts are *not* mind, neither be represented positionally (which implies *extension*), by means either of light or contact, upon an *unextended* substance. Now, this being the case, it follows that the knowledge of erect vision (erectness being one variety of position), whether the impressions upon the visual nerves are conveyed from the backs of the retinas or the iris, is instinctively produced. And as regards the objection that the term instinct is *only* a name for our ignorance, I would observe that this is but partially true, it being *in some cases* possible to perceive the existence of this power, whereas in no instance are we able to explain it. This explanation shows why no difference in the position of the eyes, when neither is removed from its normal position—not even the placing of their upper parts undermost, and vice versâ, as when any objects are viewed between the legs—makes such objects appear to any degree unerect. This condition which, physically, cannot be accounted for (it being obvious that different causes—the different situations of the impressions upon the backs of the retinas—produce the same result, the apparent erectness of objects, whatever the position of the eyes) is brought about it must be concluded, by a power which, not being understood, we are compelled to term instinct.

The cause of erect vision is one great optical difficulty; another is the cause of single vision with two eyes—a condition which, I am ready to allow, cannot be understood by the above explanation. I take it that the mind receives duality of impression only when the sensations producing the impressions are different in force; or, in other words, that when the forces are equal in strength there is unity of, and when different, duality of—what I call *degree*. Thus, when both eyes are conveyed towards any point, in which case the sensations caused by the light proceeding from it are *equal* in force (intensity), only one point is seen; whereas two images are seen of another point more or less distant,* the light proceeding from which does *not* in any case equally affect both eyes—they not being *similarly* situated with regard to the point. The phenomenon of a distant point being

* The celebrated Dr. Reid, a metaphysician much neglected by physicists, has (*Inquiry into the Human Mind on the Principles of Common Sense*) most philosophically considered these and similar phenomena; but I do not find that he has anticipated this explanation.

seen doubly has been adduced to me by a great optical authority, as being inconsistent with this explanation, he maintaining that both eyes are in the same degree affected by the last-mentioned point; but this, certainly, is not the case. It is incredible that the difference of position does *not* make a difference in the degree of the force transmitted to the visual nerves. It has also been objected that if one eye is less sensitive than the other, or imperfect, the force transmitted is different, and yet there is single vision; but I would ask how the fact of weakness proves that the force accruing from the light transmitted through the organ, is less than that produced by the light of the other eye? This is manifestly a begging of the question —a palpable *non sequitur*, inasmuch as the condition in question may be accounted for by the supposition of some *disturbance* in the passage of the light through the organ, which disturbance, be it considered, does *not* of necessity imply a diminution of its progressive force—of that it would otherwise have exerted. Moreover, the supposition of the loss of force in the way mentioned, is unphilosophical, it being known that equal forces impressed *in the same way* upon any substances, transmit the same *amount* of force, however this is varied in quality. Again, the circumstance that if a piece of glass of any colour is placed before either eye, or pieces of different colour before both, there is still single vision, cannot be brought forward in opposition to this view, there being no reason in either case for concluding that there is a difference in the strength of the forces produced. I would also add that the diversity in the impressions produced upon each eye, consequent upon their difference of position, cannot be set forth as an objection, inasmuch as the difference in the direction of the rays, by the greater angles of some and the smaller of others, does not affect the strength of the light entering either eye; and where any part is seen only by one eye, the angular compensation also prevents an irregularity, which would here be a weakness, in the strength of the light reaching the other. And it must also be observed that the fact of the corresponding points of objects *not* being represented upon the corresponding points of the retinas, is one which may at once be explained by the compensation of the rays unitedly.* The experiment of

---

* Writing concerning structural hypotheses, Sir David Brewster has said (*Life, &c. of Newton,* Edin. 1855, chap. x.): "We cannot attach any value to the invention of structural hypotheses, when the phenomena may be explained by that habitual *sympathy* of double organs with which we are so well acquainted." I am persuaded that Sir David has here been misled by a word, the term *sympathy* having no other meaning than *instinct*, which is absurd as applied to *organs*. This, manifestly, is not an explanation of the cause of single vision; and it is

Reid,* of looking at some very small and well-illuminated object, within the nearest limit of distinct vision, through three or more pin-holes in a card, within the circumference of the pupil, in which case the object is seen triply, or as many times as the number of the apertures, although very simple, is one of the greatest importance, its result being the same as that produced by combinations of lenses or semi-lenses. The plurality of impressions is caused by the fact that the light does not similarly fall upon the various parts of the retina, on which account the various currents of nerve-force produced by it are different in strength, and hence there is diversity of degree; which explanation may also, of course, be applied to an eye removed from its normal position by the finger, when any object is seen doubly. It may also be applied to all the phenomena of squinting. I am not aware that the hypothesis of M. de la Hire, that the greatest sensibility in the defective eye of a squinting person is upon one side of the centre of the retina, has in a single instance been proved correct; and if it were, the fact of single vision with one eye turned aside, could be accounted for by the part of greatest sensibility being turned towards the object, which part would receive light in a direct manner, that is, at right angles. The condition of mere eye distortion is, of course, the same as the above-mentioned artificial removal of the organ from its normal position.

It is perfectly evident, from another experiment of Reid—namely, the looking at a very small and well-illumined object beyond the nearest and within the farthest limit of distinct vision, through a pin-hole, which, being moved across the various parts of the pupil, we can see the object by the *several* rays, and this always in *the same* direction—that visible direction, the knowledge of the direction of an object, is not con-

---

surprising, a little afterwards, to meet with the following passage:—"While Dr. Reid maintains that objects appear single when the images are found upon corresponding points of the retina (all optical writers speak as if man had but *one* eye), and double in all other circumstances, he gives *no explanation whatever* of single vision." What explanation has Sir David Brewster himself produced? His remarks upon Dr. Alison's explanation are open to the same objection. I do not find that Dr. Whewell (*Phil. Induct. Scien.*, Lond. 1847) has advanced one step beyond Reid; and he has much confused the subject by speaking of the *eye* "judging" and "determining"—an obvious fallacy. We may as well apply these terms to an arm or leg. His explanation of single vision (*Paradoxes of Vision*—Single Vision: (1) Small or Distant Objects:—"as each eye judges of position, and as the two eyes judge similarly, an object will be seen in the same place by one eye and by the other, when the two images which it produces are *similarly situated* with regard to the *corresponding points* of the retina,") is that of Dr. Porterfield, thus given by Reid:—"Having the faculty of perceiving the object with each eye in its true place, we must perceive it with both eyes in the same place, and consequently must perceive it single."

* *Inquiry,* Edin. 1814, chap. vi. sec. 12, p. 268.

cluded from that of *any* of the rays proceeding from it. In fact, upon investigation, it is found that, in direct vision, any object is seen in the direction of a line perpendicular to the retina at the apex of the cone of rays, or, which is *the same thing*, and shown by Professor Rogers,* of a line bisecting these retinal lines; so that he was in error in affirming that this law is at variance with the one above (Brewster's). Nevertheless it would be absurd, indeed, manifestly so, to conclude from the results of this investigation that the significance of these lines could have been discovered apart from experience, especially as, in indirect vision, these laws are *not* applicable. I cannot understand that these laws are different to that of Reid for direct vision, "that every point of the object is seen in the direction of a right line passing from the picture of that point on the *retina*, through the centre of the eye;" this appearing to me to be only another way of describing the same line, except that the law of Reid is applicable to indirect vision—a very important difference, moreover, and one which (not, of course, as a difference, but merely as a part of its application), I do not remember that he has stated. It is also perfectly evident that the knowledge of the direction of an object by sight is altogether obtained by experience, a fact which has lately been thoroughly explained by Mr. Bain,* who has said (p. 384): "Direction is not a perception of sight alone; its very meaning precludes the supposition. It implies the locomotive or other movements that would lead us up to the object, or produce a definite change in its appearance. . . . . But without the experience of our moving organs generally, we should never know either the meaning of direction, or the fact that a certain impression on the retina implies a certain course for us to take in reference to the object. . . . . if, for example, an object were to lie in a direction inclined at 45° to the plane of its image on the retina, we should equally well become acquainted with direction; experience would connect the locomotive estimate with the visional impression, exactly as is done now;"—words which, being in every way philosophical, I have here adduced. The reader will perceive the superiority of this doctrine to that of Porterfield and Whewell, which consists of two assumptions; one, that we *do* perceive objects in their true position, and the other, that singleness of vision is the result of their being seen in the same place: neither of which propositions, but especially the first, is in any degree evident. Prior to believing the second-mentioned doctrine, the reader should demand a proof from the

---

* *American Assoc.*, E.N.P.J., Oct. 1860.

† *Senses and the Intellect*, Lond. 1855.

*mere physicist* that single vision *would* thus accrue—a proof which has not, as yet, been furnished, and which does not consist in the fact observed by Brewster, by way of a proof, that if one of the eyeballs be pressed aside two images are seen, or, I would rather say, double vision is experienced—the idea of images, and also their supposition, being intelligible only as *mental* conditions. Because, under certain physical conditions, double, and under others single, triple, or quadruple vision, is experienced, it does not follow that the respective conditions are the *causes* of the mental phenomena, although clearly it must be concluded that they are the *occasions* of them, by which I mean the conditions really producing them ; on which account, however, it should not be inferred that the *statement* of the physical conditions is any *explanation* of their mental effects, because the laws of the mind are not those of matter. This must be evident, and from it the superiority of Mr. Bain's reasoning above that of mere physicists will surely be manifest, although the example given (which extends to the mind) relates to visible direction alone. It should always be remembered that a statement of the physical causes of any phenomenon dependent upon a sense is not an explanation of its real (ultimate) cause, which, in every case, is an affection of the mind: either a blind instinct, as in upright vision, a simple affection, as in single, or a suggestion (piece of knowledge) originally obtained by experience, instinctively suggested, as in the knowledge of direction by vision. The doctrine of Sir Charles Bell,* that the constant searching motion of the eye is the means by which we become acquainted with the positions of objects, an application of the doctrine of the muscular sense, is only so far true that these motions are the indices, along with the visible rays themselves, the significance of which, as regards direction, we are by experience enabled to discover; so that this physical explanation also does not supplant experience. It is easy to see that the position of an object could no more originally, or apart from experience, be known from the motion of the eye than any of the rays proceeding from it; inasmuch as, apart from this knowledge, the laws of visual direction—that light travels in straight lines, and an object, not being visible along curves, as proved by the refraction of rays passing through the atmosphere, can only thus be seen—could not be determined ; of which laws the first, when light is seen, follows from the second.

It is a remarkable circumstance that, generally speaking, the phenomena most frequently seen are those which are the last explained. This can, in part, be accounted for by the fact that,

* *Phil. Trans.,* 1823.

as a rule, the phenomena are entirely overlooked, of which the very simple experiment of viewing any object through a pin-hole, is an example. In this case the object appears both smaller, duler, and farther off.* The small number of rays entering the eye is the cause of the dulness experienced, from which its apparent decrease of size and greater distance arises, these conditions being inferred from the former.

The idea that, in the use of the stereoscope, the successive convergence of the eyes to every part of the picture is necessary to the perception of solidity, is disproved, first, by the fact that *some* parts of the picture are *not* similar; secondly, by the fact that each *new* convergence would obliterate the impression produced by the *last;* and thirdly, by the experiments of Professor Rogers (see above reference) and those of Mr. F. August.† And the idea is also rendered very improbable by the fact, adduced as an objection by Dove, that the stereoscopic images appear solid upon the almost instantaneous illumination of the electric spark. The doctrine that solidity is caused by the pictures corresponding, more or less, with the appearances of objects as seen with both eyes, is certainly and manifestly true; but it should not from this be concluded that the appearance of relief in a solid object is thus produced, inasmuch as it appears solid when viewed only with one eye. It cannot be affirmed that any object thus seen appears solid because it is known to be so; for if any person were assured that a picture were a model, its impression would not raise the belief of its relief. The only explanation is, that a solid object appears solid because it really is so, on this account producing an impression which, in no case, results from the view of a picture, all pictorial representations being *imperfect;* in consequence of which they never appear solid, or, if so, their relief, which, of course, is in proportion to their perfection of delineation, is only exceedingly small in degree.

I shall now show how the theory of vision here propounded may be applied to the other senses. All sounds are heard

---

* I would here mention that on the 9th of November the moon, which, from where I saw it, had a dull red appearance, appeared about a foot in diameter, which increase of size I take to have been brought about with its dulness, which instinctively suggested the idea of increased distance, and hence of increased size. The difference between this case and the one mentioned in the text is this: that while the moon, the size of which cannot be realized, is really thought to be larger, the objects seen through the pin-hole, whose sizes *can* be and *are* realized, are *not* so considered; on which account their increase of dulness, which makes them appear further off, makes them also, and because of this idea of increased distance, appear smaller. The point lies in the realization (imagination) of size, the mere *knowledge* of the dimensions of any body not being of any utility in these cases.

† *Phil. Mag.*, Nov. 1860.

singly with two ears, because, mentally, there is unity of degree, which case is exactly similar to that of single vision with the two eyes. And the sense of position consequent upon touch is precisely coincident with that of position by means of the visual rays ; while the knowledge of direction from tactual impressions, like that of visible direction, is a knowledge which originally was obtained by experience, and, as in sight, is now instinctively suggested. Dr. Reid thought that sounds are heard singly because, being perfectly like and synchronous, they have nothing by which they can be distinguished; but this is not admissible as an explanation, inasmuch as *two* impressions *are* produced upon the mind, which, upon my doctrine of its constitution, are perceived together. I would not confuse myself with the word "sympathy." Moreover, the fact of want of distinction between the two impressions cannot be adduced as a reason for their singleness; for a couple of coins looked at simultaneously in any direction, through two tubes, by each eye, only one being seen by each, never appear one, because the eyes are never at the same time *precisely* similarly situated with regard to them, so that the nerve impressions are not the same in force. The knowledge of auditory, as that of olfactory direction, is no doubt originally obtained by experience, and afterwards instinctively suggested; but the fact that the direction of the causes of sounds and smells is never known when either sounds or smells are projected much out of a straight line, is a proof that this kind of experience is not possible. The sense of position does not accrue either from auditory or olfactory impressions, and why this is the case is not evident. Why does light enable us to know the position of a man, and sound not inform us of that of a bell, or smell the way in which a rose is held? By the tactual faculty, however, we are made aware of position, and this clearly by the force of instinct. When I place my hand upon my leg I am immediately conscious of its position, from which fact, seeing that the nature of the impression is not perceptibly changed by any alteration of position, I conclude that this knowledge is not obtained by experience, but instinctively. But, as regards the direction, any alteration in which causes a new experience, it may fairly be concluded that this knowledge was at first experimentally (by experience) obtained, and is now instinctively suggested. It may probably be inquired how it is that, if each hand, or any part of each hand, is placed upon the same part of the corresponding or opposite leg, or the same part of the opposite arm, *two* impressions are experienced : why, as in single vision, one only is not felt. I reply that, if the impressions were precisely similar, in which case the forces transmitted to the nerves carrying the impressions (sensations) would be the same in degree; and, if the nerves conveyed these forces to the mind, without their sameness of strength being altered, this result would accrue, the mental impressions being equal; and hence there would be singleness or unity of feeling (perception), arising from a mental condition which, in this case, would effect what I have called unity of *degree.*

---

### Art. VIII.—ON MORBID IMPULSE.*

