# Addendum to the New Theory of Vision

**Published:** 1863-04

**Authors:** 


### ADDENDUM TO THE NEW THEORY OF VISION.

Since composing the paper on this subject, I have met with Priestley's "Controversy with Price," in which my theory of the human mind is applied to the brain, an application which I do not remember to have met with in his "Disquisitions." Now, notwithstanding the great force of much that Dr. Priestley has advanced to convince the reader that the brain is the mind; or, in other words, that every mental power and moral susceptibility is the *result* of its organization of the *physical* forces accruing from the union of its various parts, I think it probable that the mind is the result of the organization of a substance, the various parts of which, though, as I have said, in no degree *mental*, yet possess certain *intermediate* powers, from the union of which the properties of mind result. I foresee some may imagine that this explanation only pushes the difficulty a little further off, thinking it just as difficult to show how the union of intermediate powers, even supposing them to allow their existence, produces a mind, as that of merely physical powers; but this conclusion would be most unphilosophical.

As regards the supposed intermediate powers, I must admit that my explanation can only be called an hypothesis; still it is a theory if only the supposition, or rather affirmation, of a substance is considered, for it is impossible but to believe that the mind *is* something (a substance). The fact that we cannot imagine intermediate powers, properties which are neither physical nor mental, cannot be brought forward to prove their non-existence; for, if what we *cannot* imagine is a criterion of what does *not* exist, or is *not* possible, then a person who had never heard of electricity or galvanism might confidently deny that any effect not attributable to ordinary friction would be produced upon him by his holding a pair of wires in his hands, on touching or approaching some particular object. The great difficulty of supposing the mind to result from the combination of certain merely *physical* forces, has led me to believe in the existence of the powers in question, which I consider to belong to some substance which is either a solid part of the brain, or is invisible, fluid, and resides in it. The anticipation of my explanation, as applied to the brain *in general*, necessitates the withdrawal of my statement, that Dr. Priestley did not answer the possible question—What part of the mind thinks?—an error, however, which any individual acquainted with his "Disquisitions" alone, might have committed.

